# The Microbial Community of Tetrodotoxin-Bearing and Non-Tetrodotoxin-Bearing Ribbon Worms (Nemertea) from the Sea of Japan

**DOI:** 10.3390/md18030177

**Published:** 2020-03-23

**Authors:** Daria I. Melnikova, Timur Yu. Magarlamov

**Affiliations:** A.V. Zhirmunsky National Scientific Center of Marine Biology, Far Eastern Branch, Russian Academy of Sciences, 690041 Vladivostok, Russia; dmelnikova@imb.dvo.ru

**Keywords:** tetrodotoxin, TTX, TTX-bearing animals, Nemertea, TTX-producing bacteria, microbiota

## Abstract

A potent marine toxin, tetrodotoxin (TTX), found in a great variety of marine and some terrestrial species, leaves intriguing questions about its origin and distribution in marine ecosystems. TTX-producing bacteria were found in the cultivable microflora of many TTX-bearing hosts, thereby providing strong support for the hypothesis that the toxin is of bacterial origin in these species. However, metagenomic studies of TTX-bearing animals addressing the whole microbial composition and estimating the contribution of TTX-producing bacteria to the overall toxicity of the host were not conducted. The present study is the first to characterize and compare the 16S rRNA gene data obtained from four TTX-bearing and four non-TTX-bearing species of marine ribbon worms. The statistical analysis showed that different nemertean species harbor distinct bacterial communities, while members of the same species mostly share more similar microbiomes. The bacterial species historically associated with TTX production were found in all studied samples but predominated in TTX-bearing nemertean species. This suggests that deeper knowledge of the microbiome of TTX-bearing animals is a key to understanding the origin of TTX in marine ecosystems.

## 1. Introduction

Tetrodotoxin (TTX) is a low molecular weight non-proteinaceous neurotoxin found in a variety of marine and some terrestrial species [1]. TTX is usually detected together with its analogues (TTXs). It causes seafood poisoning in the Indo-Pacific region. TTX poisonings associated with invasive TTX-bearing species were also reported in Europe, America, and Oceania [2]. Despite numerous data on TTX occurrence in various species including TTX-producing bacterial strains [1] and phytoplankton [3,4], the origin of the toxin and its distribution in marine ecosystems remain unclear. The hypothesis of the bacterial origin of TTX that prevails today has several weaknesses; the most serious one is that the amount of TTX produced by bacteria is small compared to its content in the respective host [2]. At the same time, all available studies of TTX-producing microflora dealt with its cultivable forms that can be maintained under laboratory conditions, while the microbial communities of TTX-bearing animals were largely ignored. However, it is not possible to determine or at least estimate the proportion of bacterial species contributing to host intoxication. Studying the microbiome of TTX-bearing animals by using metagenomic analysis provides a new approach to detecting potential TTX sources. A group of animals comprising both TTX-bearing and non-TTX-bearing species would be a good model for such studies.

Nemerteans, or ribbon worms, are mostly carnivorous marine worms belonging to a phylum of their own. TTX is detected in members of all major nemertean groups. These animals use a variety of low molecular weight neurotoxins for hunting and/or defense [5]. In ribbon worms, TTX was first found in *Lineus fuscoviridis* and *Tubulanus punctatus* in 1988 [6]. A great number of subsequent studies were dedicated to highly toxic paleonemerteans of the genus *Cephalothrix*. TTX and its analogues were found in *Cephalothrix linearis* [7,8], *Cephalothrix* sp. [9,10,11], and *Cephalothrix simula* [12,13]. Screenings for TTXs in other ribbon worms showed that the paleonemertean *T*. *punctatus*, hoplonemerteans *Quasitetrastemma stimpsoni*, *Collarenemertes bimaculata*, and *Nipponnemertes punctatula*, as well as the pilidiophores *Kulikovia alborostrata* (=*Lineus alborostratus*), *Kulikovia manchenkoi*, and *Yininemertes pratensis* contained TTXs but in much smaller concentrations than the *Cephalothrix* species [11,12,13,14]. The presence of TTX analogues in nemerteans suggests that these animals do not merely accumulate TTX but also are involved in toxin biosynthesis and metabolism. According to Yotsu-Yamashita et al. [15], the late stages of biosynthesis and metabolism of TTX and its analogues may involve oxidation routes of 5,6,11-trideoxyTTX. TTX analogues including 5-deoxyTTX, 11-deoxyTTX, and 5,6,11-trideoxyTTX itself, presumably involved in at least one oxidation route were found in *C*. *simula* [12,13] and *K*. *manchenkoi* [13]. As no conversion between TTX and its non-equilibrium analogues was detected in animals, the above mentioned oxidation reactions were supposed to proceed in TTX-producing microorganisms [16,17]. These were found in cultivable microflora of nemerteans. Members of the genera *Vibrio*, *Pseudomonas*, *Pseudoalteromonas*, and *Bacillus* isolated from both TTX-bearing and non-TTX-bearing nemerteans were claimed to synthesize TTX [12,18,19,20,21].

In this study, by using 16S rRNA gene amplicon sequencing, we characterized and compared the microbiome composition of four TTX-bearing and four non-TTX-bearing species of marine ribbon worms. The data on the microbiome of TTX-bearing animals is extremely scarce, with only one study characterizing the microbial community of two *C*. *simula* samples [12]. We are not aware of other studies addressing the microbiome of TTX-bearing animals by using metagenomic approach. Studying microbial diversity associated with TTX-bearing animals could contribute to our understanding of TTX accumulation and origin. Here, we provide for the first time original data on the microbial community of toxic and non-toxic animal species belonging to the same phylum.

## 2. Results

We sampled the microbiome of 24 ribbon worms representing four TTX-bearing and four non-TTX-bearing species (Table 1). Six species used in the current research, including *Cephalothrix simula*, *Tubulanus punctatus*, *Kulikovia alborostrata*, *Quasitetrastemma stimpsoni*, *Cerebratulus* cf. *marginatus* and *Micrura* cf. *bella*, collected from the Peter the Great Bay (Sea of Japan, Russian Far East) in July and August 2018 were tested for TTX presence or absence using high-performance liquid chromatography in combination with tandem mass spectrometry (HPLC-MS/MS) analysis in the previous study [13]. TTX analysis of *Parahubrechtia* sp. and *Hubrechtella juliae* were conducted in current research (see Section 2.4 in the Results).

By using the metagenomic approach based on the V3-V4 region of 16S rRNA gene sequencing, we characterized the total microbial community of all samples. A total of 6,328,768 reads were processed from 24 samples across 8 ribbon worm species. After pairing and filtering, this number was reduced to 1,867,843 sequences assigned to a total of 457 open-reference operational taxonomic units (OTUs) (threshold cutoff for each OTU, 97% nucleotide sequence identity using VSEARCH) spanning 29 phyla, 65 classes, 99 orders, and 142 families (Supplementary Materials). A total of 186 OTUs were identified to genus and 67 to species. The 99.87% of the OTUs belonged to the Bacteria domain; 0.09% were identified as Archaea, and 0.04% were not assigned to any taxa. The BLAST search against GenBank of unassigned OTU (757 sequences) found in one *K. alborostrata* sample, showed 83.99% identity with uncultured euryarcheotic clone OTU_340_915_195 (accession number JX021824.1) related to the environmental sample. The richest (in terms of OTUs number) and most abundant (in terms of sequence reads) prokaryote phyla included Proteobacteria (192 OTUs, 928,189 sequences (49.7%)), followed by Firmicutes (80 OTUs, 312,842 sequences (16.7%)), Bacteroidetes (56 OTUs, 197,561 sequences (10.6%)), and Actinobacteria (41 OTUs, 58,436 sequences (3.1%)). Spirochetes (6 OTUs, 49,378 (2.6%)) and Fusobacteria (4 OTUs, 56,964 (3%) phyla comprising a small number of OTUs were also relatively abundant.

Within Proteobacteria, the most abundant classes were Gammaproteobacteria (402,238 (21.5%)), Betaproteobacteria (266,151 sequences (14.2%)), Alphaproteobacteria (127,398 sequences (6.8%)), Epsilonproteobacteria (92,065 sequences (4.9%)), and Deltaproteobacteria (34,702 sequences (1.9%)). The classes Bacilli (226,600 sequences (12.1%)), Clostridia (52,415 sequences (2.8%)), and Erysipelotrichi (33,817 sequences (1.8%)) dominated within Firmicutes. In Bacteroidetes, the most abundant classes were Flavobacteria (123,755 sequences (6.63%)), Bacteroidia (49,807 sequences (2.67%)), and Saprospirae (20,098 sequences (1.1%)). The phyla Actinobacteria, Fusobacteria, and Spirochetes were dominated by the classes Actinobacteria (57,530 sequences (3.1%)), Fusobacteria (56,964 sequences (3%)), and Spirochetes (46,314 sequences (2.5%)), respectively. The most abundant orders comprising more than 1% of sequences are listed in Table 2. Among them, members of Proteobacteria (Burkholderiales, Alteromonadales, Oceanospirillales, Pseudomonadales, and Vibrionales), Firmicutes (Lactobacillales and Clostridiales), Bacteroidetes (Bacteroidales and Saprospirales), and Actinobacteria (Actinomycetales) were found in all studied nemertean species. Within Proteobacteria, the following genera were most abundant: *Methylotenera* (105,692 sequences (5.6%)), *Arcobacter* (91,132 sequences (4.9%)), *Cupriavidus* (36,439 sequences (1.9%)), *Acinetobacter* (33,360 sequences (1.7%)), *Pseudoalteromonas* (22,345 sequences (1.2%)), *Neorickettsia* (22,299 sequences (1.2%)), and *Vibrio* (7,772 sequences (0.4%)). *Leuconostoc* (50,698 sequences (2.7%)), *Tenacibaculum* (82,863 sequences (4.4%)), *Psychrilyobacter* (55,584 sequences (3%)), and *Mycobacterium* (43,583 sequences (2.3%)) dominated within *Firmicutes*, *Bacteroidetes*, *Fusobacteria*, and *Actinobacteria*, respectively.

### 2.1. Bacterial Taxonomic Distribution in Nemertean Species

**Figure 1:**
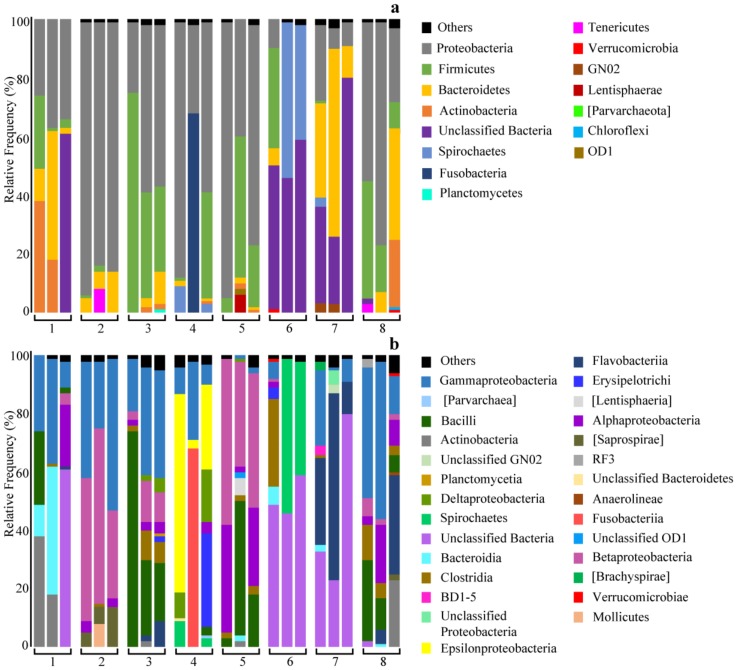
Taxonomic assignment at the phylum (**a**) and class (**b**) levels showing the relative abundance (%) of the 16S rRNA gene sequences from individual nemertean samples.

Figure 1 presents the microbial diversity in the studied nemertean species at the phylum and class levels. The proportion of microbial groups differed remarkably between nemertean species. The differences between samples from the same species were also significant. At the phylum level, the *T*. *punctatus* samples displayed the greatest similarity. They were dominated by Proteobacteria (87.3 ± 5.2%) followed by Bacteroidetes (8.5 ± 4.9%) and Firmicutes (1 ± 0.4%). One *T*. *punctatus* sample also contained a relatively great proportion of Tenericutes (7.1%). *T*. *punctatus* samples were also similar at the class level. Gammaproteobacteria, Betaproteobacteria, Alphaproteobacteria, Bacilli, and Flavobacteria were most abundant, comprising altogether 87.8 ± 5.2% of the *T*. *punctatus* microflora. Within Proteobacteria, the genus *Methylotenera* (Methylophilaceae) prevailed comprising 45.8 ± 15% of the *T*. *punctatus* microflora. Within Bacteroidetes, the family Chitinophagaceae was most abundant (8 ± 5%). The *C*. cf. *marginatus* samples were relatively similar in the taxonomic composition of their microflora. 70 ± 27.6% of their microflora was comprised of Proteobacteria, mainly Betaproteobacteria (46.2 ± 10.5%) and Alphaproteobacteria (22 ± 18.2%). The family Leuconostocaceae (22.5 ± 21.7%) was also abundant. Two *M*. cf. *bella* samples showed very similar taxonomic distribution of the microflora on both phylum and class levels including bacterial OTU not assigned to any phylum (55.2 ± 9.2%), Spirochetes (44.5 ± 7.9%), Proteobacteria (0.8 ± 0.04%), and Firmicutes (0.4 ± 0.2%). The third *M*. cf. *bella* sample differed in the absence of Spirochaetes, a greater proportion of Firmicutes (31.4%) and Bacteroidetes (7%), and the presence of Verrucomicrobia (1.2%). The *H*. *juliae* samples differed remarkably in the proportion of the dominant phyla. The abundance of Proteobacteria varied from 29% to 87.7%; Firmicutes, from 0.1% to 36%; and Fusobacteria, from 0.1% to 69.5%. In all *H*. *juliae* samples, OTUs assigned to the archaean class Parvarchaea (0.6 ± 0.7%) were found. The *K*. *alborostrata*, *C*. *simula*, and *Q*. *stimpsoni* samples were relatively similar in the microflora composition, but differed in the dominant phyla. The microbial community of *Parahubrechtia* sp. was dominated by Proteobacteria (45.3 ± 18.9%) and Firmicutes (46.7 ± 25.1%). A total of 40.1 ± 29.3% of Firmicutes was assigned to Bacilli. The *Q*. *stimpsoni* and *Parahubrechtia* sp. samples displayed the greatest microflora diversity compared to the rest nemertean species.

### 2.2. Core Microbiome of the Nemertean Species Studied

The final OTU table generated by removing mitochondrial and chloroplast sequences (but prior to rarefaction) (Supplementary Materials) was used to search for conserved OTUs across all 24 nemertean samples. Only OTUs present in more than 50% (*n* = 51) of the samples were considered (Table 3). OTU_NEM_179 assigned to the genus *Cupriavidus* (Betaproteobacteria) was found in all nemertean samples. Other most conserved OTUs, namely OTU_NEM_358 (Comamonadaceae), OTU_NEM_285 (not assigned to any phylum), and OTU_NEM_132 (OD1 phylum), were found in all nemertean species (more than 87% of the samples). Other broadly conserved sequences included members of the classes Alphaproteobacteria and Gammaproteobacteria (Proteobacteria), Clostridia and Bacilli (Firmicutes), and Actinobacteria (Actinobacteria). The comparison of the core nemertean OTUs to other GenBank sequences showed that they were taxonomically closest to environmental samples, including ones from seawater and marine sediments.

**Figure 2:**
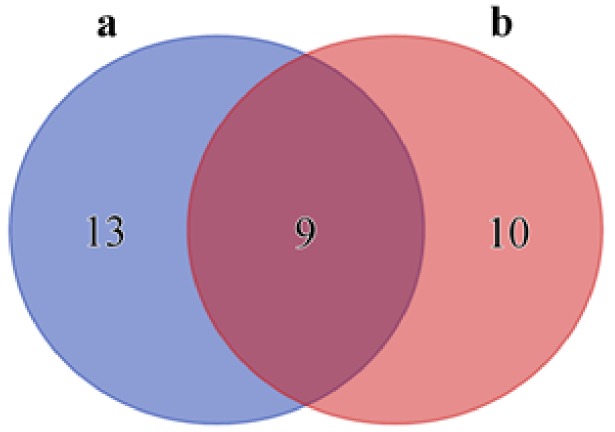
Venn diagram representing the number of stable and exclusive OTUs from a total of 32 OTUs for tetrodotoxin (TTX)-bearing (**a**) and non-TTX-bearing (**b**) nemerteans.

To evaluate differences in the conserved microflora between TTX-bearing and non-TTX-bearing nemerteans, a Venn diagram representing their core OTUs was generated (Figure 2). A total of 13 OTUs found mainly in the microflora of toxic nemerteans and 10 OTUs characteristic to non-toxic nemerteans were revealed (Table 4 and Table 5). The microflora typical of the toxic nemerteans was dominated by members of the orders Alteromonadales, Pseudomonadales, Oceanospirillales, and Vibrionales (Gammaproteobacteria). Within Gammaproteobacteria, characteristic to the non-TTX-bearing nemerteans, only Pseudomonadales and Oceanospirillales were revealed, while members of other Proteobacteria classes were also found. The core microbiome of the non-TTX-bearing nemerteans displayed a greater diversity of Firmicutes compared to the TTX-bearing ones.

### 2.3. Community Diversity of the Nemertean Microbiomes Studied

**Figure 3:**
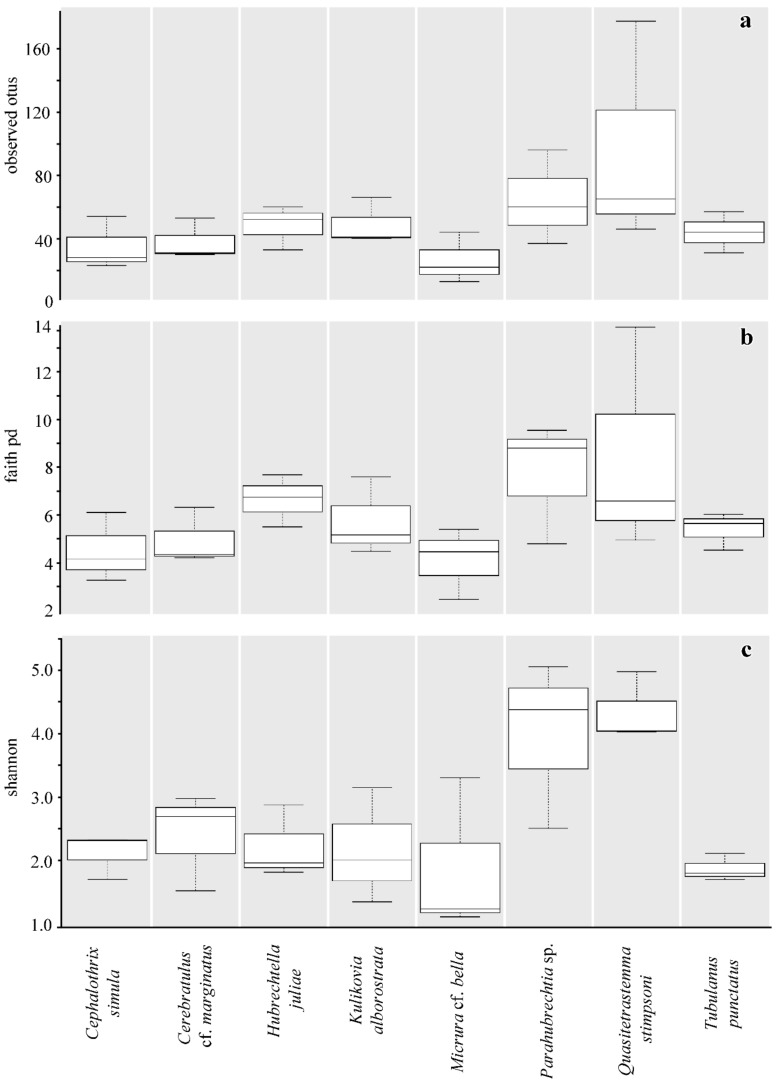
Measures of alpha diversity metrics for nemerteans microbiomes. Box-and-whisker plots comparing the numbers of observed OTUs (**a**), Faith’s phylogenetic diversity index (PD) (**b**), and Shannon diversity index (**c**). The boxes represent the interquartile range between the 75th and 25th percentiles and the internal lines the median value (50th percentile). The whiskers show the range. The ordinates: conventional units of the respective metrics.

To statistically analyze the diversity and bacterial richness of the microbial communities studied, alpha and beta diversity metrics implemented in QIIME 2 were used. The average number of observed OTUs, Shannon index, and Faith’s phylogenetic diversity index were used to determine the bacterial diversity of the nemertean species studied (Figure 3). Statistical testing showed no significant differences between the microbial communities of different nemertean species in the average number of observed OTUs (*F* = 1.57, *p* > 0.05, ANOVA; Figure 3a) and Faith’s phylogenetic diversity index (*F* = 1.46, *p* > 0.05, ANOVA; Figure 3b), while the Shannon diversity index was significantly higher in *Parahubrechtia* sp. and *Q*. *stimpsoni* compared to the other species (*F* = 4.1, *p* = 0.009, ANOVA; Figure 3c). The *Parahubrechtia* sp. and *Q*. *stimpsoni* microbiomes did not differ significantly in the Shannon diversity index (p>0.05, Student’s *t*-test). Thus, *Parahubrechtia* sp. and *Q*. *stimpsoni* had greater bacterial OTUs richness and evenness compared to the rest nemertean species.

**Figure 4:**
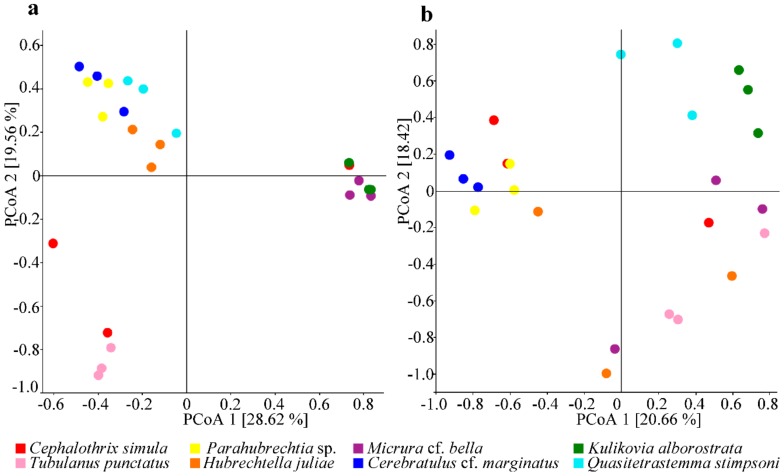
Principal coordinate analysis (PCoA) of the nemertean microbial communities based on the Bray Curtis dissimilarity distance matrix (**a**) and Jaccard distance (**b**). The plots show the distances between the communities in the samples examined (*n* = 24).

The differences in the microbiome diversity between the nemertean species studied was effectively demonstrated by principal coordinates analysis (PCoA) based on the Bray Curtis dissimilarity distance matrix (pseudo-*F* = 4.5, *p* = 0.001, PERMANOVA, 999 permutations in each test; Figure 4a). The samples from different hosts appeared to form at least three significantly different clusters, with the *T*. *punctatus* samples standing separately from the rest species, those from *M*. cf. *bella* and *K*. *alborostrata* forming another group and the other species grouping together. Interestingly, samples from the same species tended to cluster together except those from *C*. *simula*, which varied greatly in the relative abundance of OTUs. The same analysis applied to the cluster comprising *C*. cf. *marginatus*, *H*. *juliae*, *Parahubrechtia* sp., and *Q*. *stimpsoni* revealed a significant difference between these species (pseudo-*F* = 3, *p* = 0.001, PERMANOVA, 999 permutations in each test). PCoA based on Jaccard distance showed statistically significant differences between the samples from different nemerteans in the number of unique OTUs relative to the total number of OTUs (pseudo-*F* = 1.7, *p* = 0.001, PERMANOVA, 999 permutations in each test; Figure 4b). Only the samples from of *C*. cf. *marginatus*, *Parahubrechtia* sp., and *K*. *alborostrata* that formed a compact cluster were relatively similar in the membership of OTUs. The *C*. cf. *marginatus* and *Parahubrechtia* sp. samples were relatively close to two *C*. *simula* samples, assigned to the same cluster. The samples from the rest nemerteans were relatively different.

**Figure 5:**
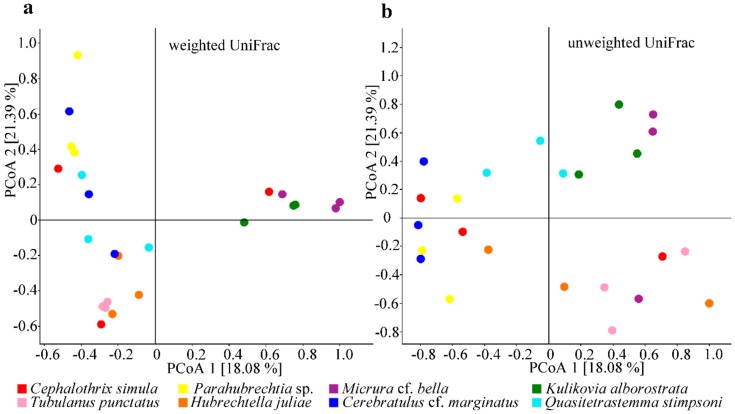
Principal coordinate analysis (PCoA) of the nemertean microbial communities based on the weighted (**a)** and unweighted (**b**) UniFrac distance matrices. The plots show the distances between the communities in the samples studied (*n* = 24).

To analyze OTU phylogeny, unweighted and weighted UniFrac distance matrices were used. PCoA based on either distance matrix revealed a significant difference between the nemertean species studied (Figure 5a; weighted UniFrac, pseudo-*F* = 3.8, *p* = 0.001, PERMANOVA, 999 permutations in each test; b. unweighted UniFrac, pseudo-*F* = 1.8, *p* = 0.001, PERMANOVA, 999 permutations in each test). By the relative abundance of observed microorganisms, based on the weighted UniFrac PCoA, the nemertean samples were clustered into two groups, with the *K*. *alborostrata* and *M*. cf. *bella* separated from the rest species (Figure 5a). By the relative occurrence of microorganisms, based on the unweighted UniFrac PCoA, significant differences were revealed both between different nemertean species and between samples from the same species (Figure 5b).

### 2.4. TTXs Analysis in Parahubrechtia sp. and Hubrechtella juliae

HPLC-MS/MS analysis of the *Parahubrechtia* sp. and *H*. *juliae* samples showed no chromatographic peaks characteristic of TTXs.

## 3. Discussion

To evaluate microbiomes, TTX-positive and TTX-negative nemerteans from different phylogenetic groups were chosen for the present study. HPLC-MS/MS analysis held in the previous research [13] revealed TTX presence in *Cephalothrix simula*, *Tubulanus punctatus*, *Kulikovia alborostrata*, *Quasitetrastemma stimpsoni* and absence of the toxin in *Cerebratulus* cf. *marginatus* and *Micrura* cf. *bella*. In the current study, no TTX was revealed in *Parahubrechtia* sp. and *Hubrechtella juliae* species.

The nemertean microflora has been poorly studied. In particular, the metagenomic approach was only used in one study characterizing two samples from toxic *C*. *simula* [12]. In the nemerteans presently studied as well as in *C*. *simula* [12], the same bacterial phyla, namely, Proteobacteria, Firmicutes, Bacteroidetes, and Actinobacteria, predominated. Some similarities were also observed in the composition of the most abundant orders; in particular, Rickettsiales, Alteromonadales, Pseudomonadales, and Vibrionales were common for all the hosts examined. On the genus level, *Alteromonas*, *Pseudomonas*, and *Vibrio* were relatively abundant both in *C*. *simula* [12] and the species presently studied. In the cultivable microflora associated with *C*. *simula* [22] and *Q*. *stimpsoni*, *K*. *alborostrata*, *H*. *juliae*, and *Malacobdella grossa* [20], members of the genera *Pseudoalteromonas*, *Pseudomonas*, *Vibrio*, *Alteromonas*, *Shewanella*, and *Bacillus* were found. The same genera were detected in the nemerteans presently examined. The predominance of Proteobacteria as well as *Bacillus* in these species is in accord with the data on the bacterial composition in marine ecosystems. This indicates that bacteria of these groups are part of the associative microflora of many marine invertebrates [23].

Though 457 OTUs were detected in the current research, only 13 core OTUs were shared among all nemerteans (Table 3). Such a small number of core OTUs may be accounted for by different feeding preferences of the nemerteans studied. The diet of ribbon worms includes semi-fluid, soft, or partly digested parts of arthropods, annelids (mostly oligochaetes and polychaetes), nematodes with a small proportion of living and dead mollusks, and fish [24,25]. The *Cephalothrix* species are also known to feed on other nemerteans and practice cannibalism [24]. The comparison of the core microbiome of the TTX-bearing and non-TTX-bearing nemerteans presently examined revealed more unique OTUs shared between the toxic species. Interestingly, members of the family Vibrionaceae, often associated with TTX production, were typical of the microflora of TTX-bearing nemerteans. Among them, *Listonella anguillarum* (=*Vibrio anguillarum*), a pathogenic bacteria causing hemorrhagic septicemia disease in marine and freshwater fish, bivalves, and crustaceans [26], was found in all toxin-bearing nemerteans, with its greatest abundance in *Q*. *stimpsoni*. Another common fish pathogen, occasionally affecting humans, *Acinetobacter johnsonii* [27] (Moraxellaceae), was found in almost all nemerteans except for *C*. *simula* and was most abundant in *K*. *alborostrata*.

The statistical analysis confirmed our previous conclusion that the nemerteans studied harbor distinct bacterial communities. Samples from the same species generally were more similar in bacterial composition compared to those from different species. The only exception was *C*. *simula*, whose three samples varied greatly in the microbial composition. *Parahubrechtia* sp. and *Q*. *stimpsoni* displayed significantly greater OTUs richness and evenness compared to the rest species. *K*. *alborostrata* and *M*. cf. *bella* were more similar in the relative abundance of observed OTUs as revealed by both Bray Curtis and weighted UniFrac PCoA analysis. *Parahubrechtia* sp., *C*. cf. *marginatus*, *Q*. *stimpsoni*, and *H*. *juliae* were assigned to the same cluster by Bray Curtis PCoA indicating their similarity in the microbiome composition. These results do not clearly demonstrate the relationship between hosts phylogeny and bacterial community. Microbial communities usually show more similarity between closely related hosts than between distant ones [28]. Among marine invertebrates, it was shown for sponges in a number of studies [29,30,31]. In the present study, *K*. *alborostrata* and *M*. cf. *bella*, both from the family Lineidae, had similar microbial communities. *Parahubrechtia* sp. and *T*. *punctatus* (Tubulanidae) on the one hand and *C*. cf. *marginatus* and *K*. *alborostrata* (Lineidae) on the other displayed sizeable dissimilarity in their microbiomes.

To date, members of 31 bacterial genera claimed in TTX production were isolated from various TTX-bearing organisms, as well as marine and freshwater environments [1]. Carroll et al. [18] were the first to report the relationship between the presence of *Vibrio* bacteria and TTX synthesis in seven species of the British nemerteans. It is worth noting that although the genus *Vibrio*, widespread in marine ecosystems, was commonly reported as the main bacterial TTX-producer, its ability to produce the toxin was questioned. Strand et al. [32] showed that using liquid chromatography-mass spectrometry (LC-MS) and bioassay for TTX detection in bacterial and nemertean samples might lead to false-positive results and should be supplemented with tandem mass spectrometry (MS/MS). The authors revealed a compound produced by *Vibrio alginolyticus* isolated from the ribbon worm *Lineus longissimus*. It was similar to TTX in molecular weight but differed from it in retention time and MS/MS fragmentation pattern and exhibited no toxic effect. However, recently, Turner et al. [12] confirmed the TTX production for *V. alginolyticus* isolated from the TTX-negative nemertean *Tubulanus annulatus* using LC-MS/MS. The present study showed that *Vibrio* is abundant in the nemertean microflora and is predominant in TTX-bearing species. *V*. *alginolyticus* was not found in the nemerteans presently examined. However, as was mentioned above, *L*. (=*Vibrio*) *anguillarum*, also claimed to produce TTX [33], was revealed in all TTX-bearing nemerteans. Interestingly, other members of Gammaproteobacteria, known to produce TTX, including *Pseudomonas*, *Shewanella*, *Alteromonas*, *Marinomonas*, and *Pseudoalteromonas*, were found in the majority of the nemertean species presently studied and were most abundant in TTX-bearing *K*. *alborostrata* and *Q*. *stimpsoni*. Recently, Turner et al. [12] isolated *Pseudomonas luteola*, capable of TTX synthesis, from *C*. *simula*. By using anti-TTX immunocytochemistry, Melnikova et al. [20] showed that the *Pseudoalteromonas* sp. strain 1942 isolated from *H*. *juliae* displayed TTX immunoreactivity. The bacterial strain *Bacillus* sp. 1839 isolated from *C*. *simula* was also reported to produce TTX first by using confocal laser scanning microscopy with polyclonal anti-TTX antibodies [19], and later on, by means of LC-MS/MS analysis [21]. In the present study, *Bacillus* species were more abundant in the microflora of non-TTX-bearing nemerteans, especially, in *H*. *juliae* and *Parahubrechtia* sp. and in TTX-bearing *Q*. *stimpsoni*. Moreover, the similarity search between a TTX-producing *Bacillus* sp. 1839 16s rRNA sequence and the present data revealed 16 sequences with 97% similarity with this strain in a *Q*. *stimpsoni* sample. We expected *C*. *simula*, the most toxic nemertean known to date, to display the greatest abundance of bacteria historically associated with TTX production. However, these bacteria occurred in all nemerteans presently studied with the predominance in the TTX-bearing species. In concordance with this, TTX-producing bacteria were found in TTX-negative nemerteans [12,21]. Nevertheless, one may conclude that TTX-positive nemerteans tend to accumulate more TTX-producing strains in their microflora compared to TTX-negative ones.

## 4. Materials and Methods 

### 4.1. Sample Collection

Samples of *Kulikovia alborostrata* (Takakura, 1898), *Micrura* cf. *bella* (Stimpson, 1857), *Cerebratulus* cf. *marginatus* Renier, 1804, *Cephalothrix simula* (Iwata, 1952), *Tubulanus punctatus* Takakura, 1898, *Quasitetrastemma stimpsoni* (Chernyshev, 1992), *Parahubrechtia* sp., and *Hubrechtella juliae* (Chernyshev, 2003) were collected in July and August 2019 in the Spokoynaya (42.7090N, 133.1809E) and Vostok (42.9021N, 132.7385E) Bays of the Peter the Great Bay (Sea of Japan, Russian Far East). All nemertean specimens were kindly identified by Dr. Alexey V. Chernyshev, an expert in the nemertean zoology. Representatives of the majority of the species studied were collected in rhizoids of the brown algae *Saccharina* sp. at a depth of up to 1 m. *Parahubrechtia* and *Hubrechtella* were collected by trawling in mud at a depth of 5–7 m, and *Cerebratulus*, in sandy mud at a depth of up to 2 m. The water temperature in the collection sites was 17–21.7 °C. For 16s rRNA sequencing analyses, three individuals of each species were sampled. The samples were immediately transported to the laboratory, rinsed with sterilized seawater, placed in 100% ethanol, and stored at −20 °C until processing. For toxin testing, rinsed specimens were frozen at −20 °C without ethanol preservation.

### 4.2. DNA Extraction and 16S rRNA Sequence Processing

Ethanol-preserved nemertean samples of the *Kulikovia*, *Micrura*, *Cerebratulus*, *Cephalothrix*, and *Tubulanus* species were dissected into 20 mg cross sections used for DNA extraction. Specimens of the *Parahubrechtia*, *Hubrechtella*, and *Quasitetrastemma* species, taken for the same purpose, were not dissected due to their small size. Sections or whole worms were homogenized in a 1.5 mL Eppendorf LoBind tube containing 350 μL of lysis buffer with Proteinase K using a hand homogenizer for 3 min. Using an E.Z.N.A. Mollusc DNA Kit (Omega Bio-tek, Norcross, GA, USA), total genomic DNA was extracted from each sample. The extraction was performed following the manufacturer’s protocol with one exception: vortexing steps were replaced by gentle hand rotation of the samples. DNA purity and concentration were assessed using an UV5Nano spectrophotometer (Mettler Toledo, USA). The amount of DNA per sample varied from 39.7 to 509.8 ng/μL. All samples were stored at −20 °C until used. 

For metagenomic analysis based on V3-V4 region 16S rRNA gene amplicons, total genomic DNA was amplified using Herculase II Fusion DNA Polymerase Nextera XT Index Kit V2 and sequenced on an Illumina Miseq platform with 2 × 301 bp paired-end reads (Macrogen Inc., Seoul, South Korea). The demultiplexing and quality control of the Illumina MiSeq dataset was processed by the Macrogen Inc.

### 4.3. Sequence Processing and Taxonomic Assignment

The quality of the reads was filtered using BBDuk software to remove Illumina adapters and artifacts (parameters for the pass: “ktrim = r k = 27 qtrim = rl trimq = 20 minoverlap = 24 minlength = 150”; http://jgi.doe.gov/data-and-tools/bb-tools/). Bioinformatics analysis was conducted using the Quantitative Insights into Microbial Ecology toolkit (QIIME) 2 version 2019.7 (https://qiime2.org) with proper built-in plugins [34]. The VSEARCH algorithm [35] was applied to merge the forward and reverse reads. Merged reads with an overlap length below 400 bp or over 570 bp were discarded. The quality-filtered sequences were dereplicated and combined into a single FASTA file for open-reference operational taxonomic unit (OTU) picking at 97% sequence identity. A representative sequence from each OTU was aligned against the Greengenes core set (database version 13.8) [36] using the feature-classifier plugin from QIIME2 with the classify-sklearn method [37,38]. The sequences that were not successfully classified by the Greengenes classifier were manually BLAST searched to specify taxonomy. Chimeric sequences were removed using the VSEARCH. Chloroplast and mitochondria sequences were excluded from downstream analyses. Core microbiomes were identified by the presence of an OTU in >50% (*n* = 51) of the nemertean samples.

Sequence files and metadata for all samples used in this study have been deposited in NCBI Sequence Read Archive repository under BioProject PRJNA604891 (accession numbers SRR11027358-SRR11027381).

### 4.4. Statistical Analysis

Alpha and beta diversity metrics were calculated using QIIME 2 [34] on a rarefied dataset to prevent statistical artifacts due to different sequencing depths. Alpha diversity was measured with the number of observed OTUs, Shannon diversity index, and Faith’s phylogenetic diversity index. The sampling depth was set to 14590. The statistical significance of between-species differences in alpha diversity was evaluated using one-way analysis of variance (ANOVA) at *p* < 0.05. Beta diversity was measured with the Jaccard distance, Bray–Curtis distance, weighted and unweighted UniFrac distances, and clustered by PCoA. The statistical significance of sample clustering on these distance metrics was estimated using permutational multivariate analysis of variance (PERMANOVA) test at *p* < 0.05. Only OTUs found in at least 4 samples were included in the beta diversity analyses. The sampling depth was set to 7400.

### 4.5. Toxin Testing in Parahubrechtia sp. and Hubrechtella juliae

Extracts of *Parahubrechtia* sp. and *H*. *juliae* were prepared as described in Vlasenko et al., 2018 [13]. Briefly, samples were homogenized in 0.1% acetic acid in 70% methanol solution 1:10 (vol/vol) and ultrasonically treated. The homogenates were centrifuged, the supernatants were taken out and evaporated, and the resulting precipitates were dissolved in 0.1% aqueous acetic acid solution to the liquid-solid ratio of 1 mL/g, and filtered through centrifugal concentrators. The extracts were analyzed for tetrodotoxin (TTX) and its associated analogues (TTXs) by using HPLC-MS/MS following Bane et al. (2016) with modifications described in Vlasenko et al., 2018 [13]. TTX concentration was calculated using the calibration curve of standard TTX solution series (Alomone Labs Ltd, Jerusalem, Israel). The concentration of TTX analogues was calculated following the procedure proposed by Chen et al. [39]. The toxin detection criteria were the S/N ratio of the precursor MRM transition peak > 3, the relative intensity of the fragment ion peak > 4%, the order of toxins elution following Bane et al. [40]. The method was validated using standard TTX solutions in MRM mode. The linearity range varied from 0.6 to 100 ng/mL, the recovery range from 1 to 100 ng/mL of TTX was 98.4%, the LoQ was 0.6 ng/mL, the limit of detection (LoD) was 0.2 ng/mL, and the relative standard deviation varied within 4.5–14.6%.

## 5. Conclusions

The present study is the first to characterize the composition of the bacterial communities of TTX-bearing and non-TTX-bearing marine ribbon worms belonging to different taxa. The nemerteans studied proved to differ considerably in the microbiome composition. We also addressed the relationship between the presence of TTX-producing bacteria and TTX content in nemerteans, and we concluded that the TTX-bearing nemerteans presently studied tend to have more TTX-producing strains in their microflora. The present study provides the basis for further research on the microbial ecology and TTX distribution in Nemertea. Identifying TTX source(s) in one group of marine invertebrates may shed light on the TTX origin in marine ecosystems as a whole.

## Figures and Tables

**Table 1:** The nemertean species used in the present study.

Nemertean Species	TTXs Presence	Reference
Palaeonemertea		
*Cephalothrix simula* (Iwata, 1952)	+	[13]
*Tubulanus punctatus* Takakura, 1898	+	[13]
*Parahubrechtia* sp.	−	Present study
Pilidiophora		
*Hubrechtella juliae* (Chernyshev, 2003)	−	Present study
*Cerebratulus* cf. *marginatus* Renier, 1804	−	[13]
*Micrura* cf. *bella* (Stimpson, 1857)	−	[13]
*Kulikovia alborostrata* (Takakura, 1898)	+	[13]
Hoplonemertea		
*Quasitetrastemma stimpsoni* (Chernyshev, 1992)	+	[13]

+ — TTXs are found; − — TTXs are not found.

**Table 2:** The most abundant bacterial orders identified in nemertean hosts.

Phylum	Class	Order	% of All Sequences	Found in Nemertean Species *
Proteobacteria	Alphaproteobacteria	Rickettsiales	1.8	1,2,3,5,8
	Betaproteobacteria	Burkholderiales	2.3	All
		Methylophilales	5.7	1,2,3,4,5,8
	Deltaproteobacteria	Sva0853	1.3	4
	Gammaproteobacteria	Alteromonadales	1.3	All
		Oceanospirillales	3.3	All
		Pseudomonadales	2.4	All
		Vibrionales	6	All
	Epsilonproteobacteria	Campylobacterales	4.9	2,3,4,5,6,7,8
Firmicutes	Bacilli	Lactobacillales	11.9	All
	Clostridia	Clostridiales	2.8	All
	Erysipelotrichi	Erysipelotrichales	1.8	1,2,3,4,6,8
Bacteroidetes	Bacteroidia	Bacteroidales	2.7	All
	Flavobacteria	Flavobacteriales	6.6	1,2,3,4,5,7,8
	Saprospirae	Saprospirales	1	All
Actinobacteria	Actinobacteria	Actinomycetales	3	All
Fusobacteria	Fusobacteria	Fusobacteriales	3	2,4,8

* Nemertean species: 1—*Cephalothrix simula*; 2—*Tubulanus punctatus*; 3—*Parahubrechtia* sp.; 4—*Hubrechtella juliae*; 5—*Cerebratulus* cf. *marginatus*; 6—*Micrura* cf. *bella*; 7—*Kulikovia alborostrata*; 8—*Quasitetrastemma stimpsoni*.

**Table 3:** Core bacterial operational taxonomic units (OTUs) present in >50% of nemertean samples (*n* = 51).

OTU ID	Taxonomy According to Greengenes	% of Nemertean Samples	Found in Nemertean Species *
OTU_NEM_285	Kingdom Bacteria	92	All
OTU_NEM_132	Kingdom Bacteria; phylum OD1	87.5	All
OTU_NEM_306	Kingdom Bacteria; phylum Actinobacteria; class Actinobacteria; order Actinomycetales; family Corynebacteriaceae; *Corynebacterium*	58	1,2,3,4,5,6,8
OTU_NEM_332	Kingdom Bacteria; phylum Proteobacteria; class Alphaproteobacteria; order Rhizobiales; family Bradyrhizobiaceae	62.5	All
OTU_NEM_179	Kingdom Bacteria; phylum Proteobacteria; class Betaproteobacteria; order Burkholderiales; family Oxalobacteraceae; *Cupriavidus*	100	All
OTU_NEM_358	Kingdom Bacteria; phylum Proteobacteria; class Betaproteobacteria; order Burkholderiales; family Comamonadaceae	92	All
OTU_NEM_454	Kingdom Bacteria; phylum Proteobacteria; class Gammaproteobacteria; order Pseudomonadales; family Moraxellaceae; *Acinetobacter*	71	1,2,3,4,5,7,8
OTU_NEM_327	Kingdom Bacteria; phylum Proteobacteria; class Gammaproteobacteria; order Pseudomonadales; family Moraxellaceae; *Acinetobacter johnsonii*	62.5	1,2,3,4,6,7,8
OTU_NEM_396	Kingdom Bacteria; phylum Proteobacteria; class Gammaproteobacteria; order Vibrionales; family Vibrionaceae	62.5	1,2,4,6,7,8
OTU_NEM_95	Kingdom Bacteria; phylum Proteobacteria; class Gammaproteobacteria; order Enterobacteriales; family Enterobacteriaceae	75	All
OTU_NEM_126	Kingdom Bacteria; phylum Firmicutes; class Clostridia; order Clostridiales; family Lachnospiraceae	66.7	All
OTU_NEM_315	Kingdom Bacteria; phylum Firmicutes; class Clostridia; order Clostridiales; family Lachnospiraceae; [*Ruminococcus*]	54	1,2,4,6,7,8
OTU_NEM_231	Kingdom Bacteria; phylum Firmicutes; class Bacilli; order Lactobacillales; family Lactobacillaceae; *Lactobacillus*	58	1,2,3,4,5,7,8

* Nemertean species: 1—*Cephalothrix simula*; 2—*Tubulanus punctatus*; 3—*Parahubrechtia* sp.; 4—*Hubrechtella juliae*; 5—*Cerebratulus* cf. *marginatus*; 6—*Micrura* cf. *bella*; 7—*Kulikovia alborostrata*; 8—*Quasitetrastemma stimpsoni*.

**Table 4:** Unique bacterial OTUs present in >50% of TTX-bearing nemerteans (*n* = 51).

OTU ID	Taxonomy According to Greengenes	% of Nemertean Samples	Found in Nemertean Species *
OTU_NEM_34	Kingdom Bacteria; phylum Actinobacteria; class Actinobacteria; order Actinomycetales; family Propionibacteriaceae; *Propionibacterium acnes*	58	All
OTU_NEM_263	Kingdom Bacteria; phylum Bacteroidetes; class Flavobacteria; order Flavobacteriales; family Flavobacteriaceae	58	All
OTU_NEM_288	Kingdom Bacteria; phylum Proteobacteria; class Gammaproteobacteria	67	1,2,4
OTU_NEM_102	Kingdom Bacteria; phylum Proteobacteria; class Gammaproteobacteria; order Alteromonadales; family Alteromonadaceae; *Alteromonas*	67	All
OTU_NEM_158	Kingdom Bacteria; phylum Proteobacteria; class Gammaproteobacteria; order Alteromonadales; family Colwelliaceae	67	2,3,4
OTU_NEM_446	Kingdom Bacteria; phylum Proteobacteria; class Gammaproteobacteria; order Alteromonadales; family Moritellaceae; *Moritella*	58	2,3,4
OTU_NEM_19	Kingdom Bacteria; phylum Proteobacteria; class Gammaproteobacteria; order Alteromonadales; family Psychromonadaceae; *Psychromonas*	58	2,3,4
OTU_NEM_327	Kingdom Bacteria; phylum Proteobacteria; class Gammaproteobacteria; order Pseudomonadales; family Moraxellaceae; *Acinetobacter johnsonii*	83	All
OTU_NEM_114	Kingdom Bacteria; phylum Proteobacteria; class Gammaproteobacteria; order Oceanospirillales; family Oceanospirillaceae; *Marinomonas*	58	2,3,4
OTU_NEM_2	Kingdom Bacteria; phylum Proteobacteria; class Gammaproteobacteria; order Vibrionales; family Pseudoalteromonadaceae; *Pseudoalteromonas*	67	All
OTU_NEM_396	Kingdom Bacteria; phylum Proteobacteria; class Gammaproteobacteria; order Vibrionales; family Vibrionaceae	92	All
OTU_NEM_45	Kingdom Bacteria; phylum Proteobacteria; class Gammaproteobacteria; order Vibrionales; family Vibrionaceae; *Listonella anguillarum*	67	All
OTU_NEM_315	Kingdom Bacteria; phylum Firmicutes; class Clostridia; order Clostridiales; family Lachnospiraceae, [*Ruminococcus*]	67	All

* Nemertean species: 1—*Cephalothrix simula*; 2—*Tubulanus punctatus*; 3—*Kulikovia alborostrata*; 4—*Quasitetrastemma stimpsoni*.

**Table 5:** Unique bacterial OTUs present in >50% of non-TTX-bearing nemerteans (*n* = 51).

OTU ID	Taxonomy According to Greengenes	% of Nemertean Samples	Found in Nemertean Species *
OTU_NEM_306	Kingdom Bacteria; phylum Actinobacteria; class Actinobacteria; order Actinomycetales; family Corynebacteriaceae; *Corynebacterium*	67	All
OTU_NEM_14	Kingdom Bacteria; phylum Bacteroidetes; class Saprospirae; order Saprospirales; family Chitinophagaceae; *Sediminibacterium*	58	All
OTU_NEM_103	Kingdom Bacteria; phylum Proteobacteria; class Alphaproteobacteria; order Rhizobiales; family Xanthobacteraceae	58	All
OTU_NEM_287	Kingdom Bacteria; phylum Proteobacteria; class Epsilonproteobacteria; order Campylobacterales; family Campylobacteraceae; *Arcobacter*	58	All
OTU_NEM_98	Kingdom Bacteria; phylum Proteobacteria; class Gammaproteobacteria; order Pseudomonadales; family Pseudomonadaceae; *Pseudomonas nitroreducens*	58	All
OTU_NEM_94	Kingdom Bacteria; phylum Proteobacteria; class Gammaproteobacteria; order Oceanospirillales; family Endozoicimonaceae	58	2,3,4
OTU_NEM_156	Kingdom Bacteria; phylum Firmicutes; class Clostridia; order Clostridiales	58	All
OTU_NEM_134	Kingdom Bacteria; phylum Firmicutes; class Bacilli; order Lactobacillales	58	2,3,4
OTU_NEM_138	Kingdom Bacteria; phylum Firmicutes; class Bacilli; order Lactobacillales; family Leuconostocaceae	58	2,3,4
OTU_NEM_208	Kingdom Bacteria; phylum Firmicutes; class Bacilli; order Lactobacillales; family Leuconostocaceae; *Leuconostoc*	58	2,3,4

* Nemertean species: 1—*Micrura* cf. *bella*; 2—*Cerebratulus* cf. *marginatus*; 3—*Parahubrechtia* sp.; 4—*Hubrechtella juliae*.

## Supplementary Materials

The following are available online at https://www.mdpi.com/1660-3397/18/3/177/s1, OTUs table by sample and nemertean host. The table presents OTU abundance per sample, reference sequence, and taxonomic affiliation according to the Greengenes database.
